# A Nonparametric Shape Prior Constrained Active Contour Model for Segmentation of Coronaries in CTA Images

**DOI:** 10.1155/2014/302805

**Published:** 2014-04-01

**Authors:** Yin Wang, Han Jiang

**Affiliations:** ^1^State Key Laboratory of Virtual Reality Technology and Systems, Beihang University, Beijing 100191, China; ^2^College of Astronautics, Nanjing University of Aeronautics and Astronautics, Nanjing 210016, China

## Abstract

We present a nonparametric shape constrained algorithm for segmentation of coronary arteries in computed tomography images within the framework of active contours. An adaptive scale selection scheme, based on the global histogram information of the image data, is employed to determine the appropriate window size for each point on the active contour, which improves the performance of the active contour model in the low contrast local image regions. The possible leakage, which cannot be identified by using intensity features alone, is reduced through the application of the proposed shape constraint, where the shape of circular sampled intensity profile is used to evaluate the likelihood of current segmentation being considered vascular structures. Experiments on both synthetic and clinical datasets have demonstrated the efficiency and robustness of the proposed method. The results on clinical datasets have shown that the proposed approach is capable of extracting more detailed coronary vessels with subvoxel accuracy.

## 1. Introduction

Reliable and correct vessel segmentation is a crucial and fundamental step towards the designing of computer-aided systems in diagnosis of vascular diseases. Despite intensive amounts of research efforts dedicated to the development of semi- or fully automated algorithms for extraction of vascular structures in medical images, the task of defining correct boundaries of vascular structures is still challenging, because of the complex geometrical structure of vessels and pathological lesions that could introduce undesired artifacts. Besides, images features may change dramatically in terms of image modalities and anatomical applications.

Existing methods can be generally classified into two categories, namely, tracking based algorithms and deformable model based approaches, respectively. Vessel segmentation using tracking based techniques generally begins with a number of starting points, also known as seeds, and then the neighboring pixels are added to the foreground objects in terms of predefined criteria. Among them, minimum path based methods [1–4] are particularly popular in the context of vessel segmentation. Wink and his coworkers [5] proposed the application of the filter response from vessel enhancement algorithms [6–8] as the minimum path energy for extraction the vessels in MRA images. The work was further adopted in [9] to improve the performance of their method in the presence of varying vessel diameters and junctions, by using the scale factor of vessel enhancement filters as an additional parameter. However the vesselness measurement, derived from second order derivative information, is obtained based on single branch vessel model. Thus it could lead to erroneous segmentation in case where such assumption cannot be hold, for example, in the proximal areas of aneurysms, stenosis, and bifurcations. Based on local intensity profiles of vessels, Kaftan et al. [10] proposed a “medialness” vesselness measurement as the minimum path energy, which improves the performance of the proposed algorithm with respect to local intensity variations. The method was further improved in [11], where the authors proposed calculating the medialness metric only in selected pixels in terms of current segmentation, and thus the efficiency of their algorithm has been greatly increased. Hua and Yezzi [12] proposed modeling vascular structures as a succession of spheres to simultaneously detect both the vessel centerline and associated boundaries. In their method, the regional statistics are only derived from the boundaries of the spheres, thus improving the robustness of the segmentation respect to image noise. In the same research line, Antiga et al. [13] proposed a surface domain method by finding the locus of centers of maximal spheres inscribed into the tubular structures using Voronoi diagram. Florin and his colleagues [14] modeled vessels as a series of elliptical cross-sections and detected the vessels based on particle filters. Compared to the previously discussed deterministic approaches, their algorithm is developed based on stochastic filters, which provides additional statistical properties about the resulting segmentation. Following their seminal work, Schaap et al. [15] applied a succession of short tubes to model the vessel segments, which greatly enhances the robustness and accuracy of particle filter based methods.

Active contour models [16], known as snakes, represent one of the most popular model based methods in medical images processing communities. The algorithm, in general, is performed by iteratively deforming a contour/surface until the associated contour energy is minimized. The active contour energy is usually determined by its shape and image-driven features, such as image edges. After the pioneered work in [17, 18], a great deal of researches have been dedicated to develop varieties of image features to drive the active contour to the desired boundaries. Yang et al. [19] proposed incorporating active contour segmentation into a Bayesian probabilistic framework, where the image-driven energy in their method is redefined by posterior probabilities. As a contrast to early works using image edges to formulate image-based energies, region based image features, relying on global information derived from image regions, are more robust to noisy and inhomogeneous gradients. A well-known region based active contour model was reported by Chan and Vese (known as CV model) [20], who proposed a simplified solution to Mumford-Shah functional minimization problem based on the framework of level sets. Following their seminar work, Rousson et al. proposed more robustly model regional statistics by using both mean and variance information [21]. Lecellier et al. improve the accuracy and robustness of the modeled statistics by using the entire family of exponential functions [22]. Localized active contours [23–25] have been introduced to improve the performance of the active contours in the presence of varying brightness across the image. Lankton and Tannenbaum, [26] proposed the use of different metrics to measure the similar intensity distributions derived from image regions. Despite the benefits introduced by measuring the regional statistics locally, the selection of the appropriate scale poses new difficulties in these models. If the scale is selected to be small, the intensity density would be estimated based on a small number of pixels; this would increase the capability of the active contour to identify weak edges, but it also makes the active contour be sensitive to image noise and initialization. On the other hand, when a too large window size is used, the intensity distribution is calculated based on “global” information, which cannot take the advantage of localized techniques.

The leakage problem is often encountered in medical image segmentation when only intensity features are utilized, since boundaries between different objects cannot be always defined by intensities. Shape priors [27, 28], generally defined based on a training set, have been introduced as an additional hard constraint to improve the segmentation when the objects to be segmented exhibit similar appearances to the ones present in the training sets. Reliable shape priors, however, are in general difficult to determine in practice due to the high interpatient variability of the vessel geometries and the limited availability of training datasets. In addition, parameterization of shape features from training set and determination of their correspondences are also challenging. To address these problems, Nain et al. [29] incorporate a soft shape prior into the framework of active contours, which allows the shape information to be directly derived from the current segmentation. In their work, the shape descriptor was defined as a ball structure centered on each point along the active contour, and the likelihood of current segmentation to the vascular structures is determined by measuring the percentage of pixels belonging to both the ball and the object. The leakage can be therefore detected when output of the shape descriptor is high. By the introduction of the shape descriptor, the potential leakage would be penalized during contour evolution. Yet, their proposed shape descriptor is unable to discriminate vessel bifurcations from leakage areas, which leads to undesired gaps in the vicinity of these regions.

The contributions of the present work are twofold. Firstly, we propose an active contour energy functional for vessel segmentation with an adaptive scale selection scheme, which allows the localized kernel varying in an automated fashion based on prior knowledge regarding the histogram distribution of the CTA images. The proposed adaptive scale selection scheme improves the performance of the localized active contour model for weak edges. Secondly, we propose coupling a nonparametric shape constraint into the active contour framework, which prevents the contour leak into neighboring regions and improves the segmentation results. Followed by this introduction, we provide great details about the proposed method in Section 2. This is followed by the presentation of the experimental results on both synthetic and clinical datasets, demonstrating the efficiency of the proposed approach. The conclusions of this work and future research are presented in the final section.

## 2. Materials and Methods

**Figure 1:**
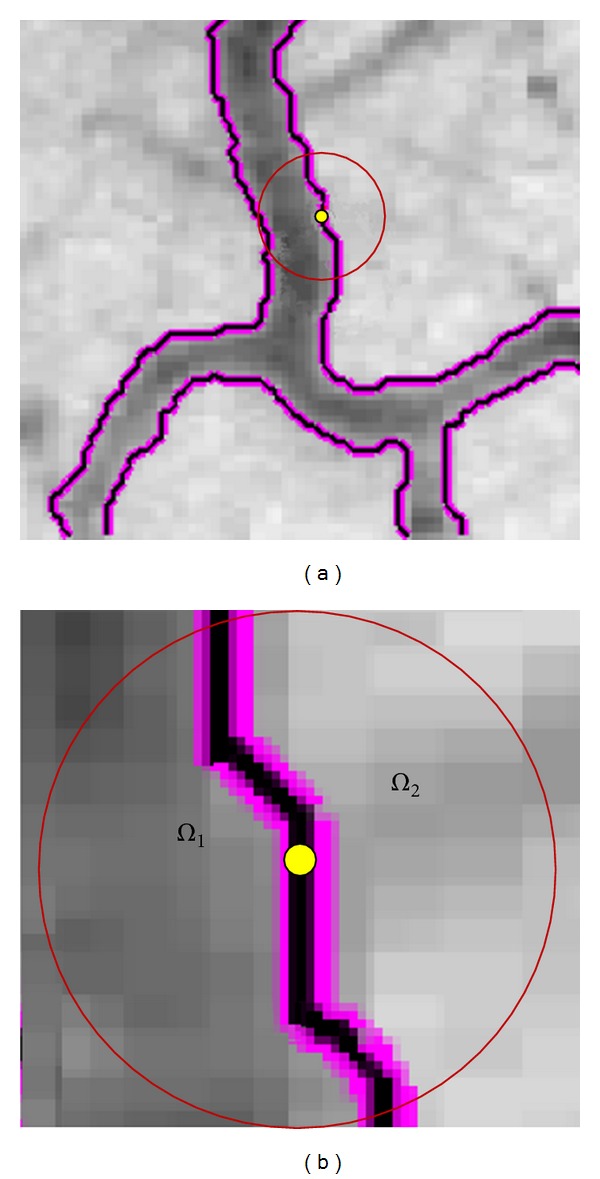
Synthetic image illustrates the active contour model based on localised regional statistics. (a) A randomly selected centre point is shown in yellow from the active contour; red circle defines a localised image. (b) The zoomed-in image of circular neighbourhood defined in (a). Note that we neglect the pixels outside of the local window.

Let *Ω*_**x**_ denote a local image with a radius *r* centered at **x** on the active contour *C*(**x**). As depicted in Figure 1, the localized image, *Ω*_**x**_, can be partitioned into two subregions by the active contour. If we assume that the intensity distribution within these image regions can be approximated by a two-parameter Gaussian function, the probability of a pixel being classified as belonging to the region *Ω*_*i*_ can be calculated as follows:

(1) Pi=P(I(y) ∣ y∈Ωi∩Ωx)=12πσiexp⁡(−(μi−I(y))22σi2),

where {*Ω*_*i*_ | *i* = 1,2} represent the regions inside and outside the contour, respectively. *I*(**y**) denotes the image intensity values at position **y**, *μ*_*i*_ and *σ*_*i*_ are the mean and the variance derived from region *Ω*_*i*_, respectively. It should be noted that we use **x** and **y** as two independent spatial variables in this paper.

### 2.1. Adaptive Scale Selection Scheme

As discussed in previous section, the localized active contours, using a constant window size, may lead to erroneous segmentation when local image contrast is weak. To remedy this problem, we present an adaptive scale section scheme based on the global histogram distribution of the input image, which allows for the determination of the appropriate scale for each point along the active contour in an automated fashion. When local image contrast is low, we use a relatively smaller window size to increase the sensitivity of the active contour in detecting weak edges; on the other hand, a larger localized image would be considered when image contrast is relatively higher, which improves the robustness of the active contour to image noise.

**Figure 2:**
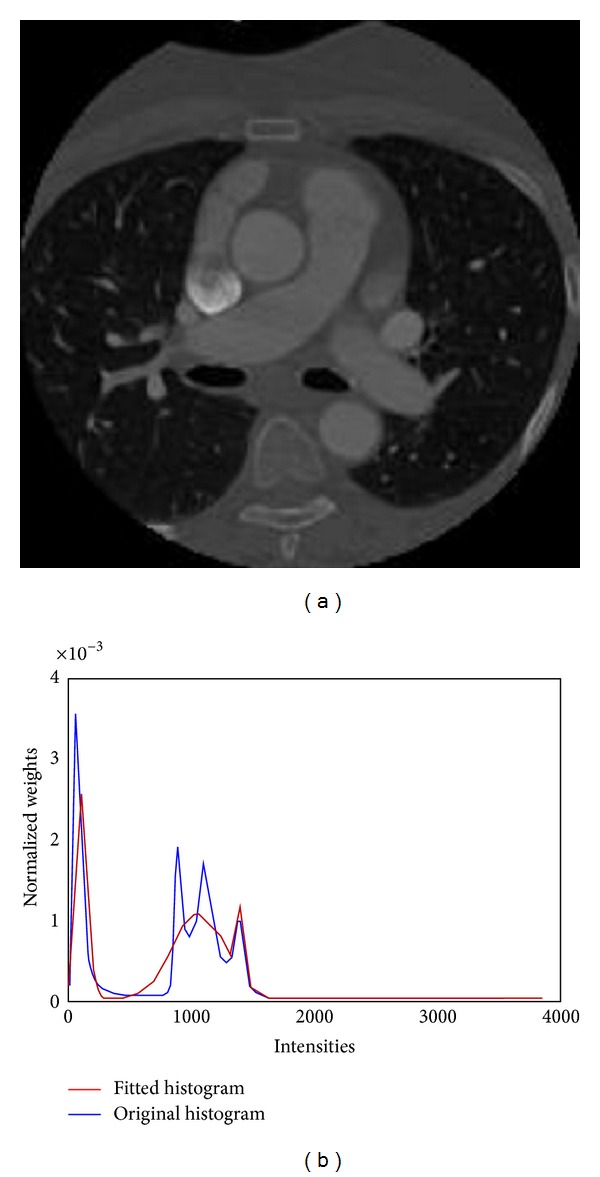
The histogram distribution of CTA image. (a) An axial slice of the CTA image randomly picked from the datasets. (b) The intensity distribution (blue) and the fitted mixture model (red) of the CTA image.

Figure 2 shows a typical histogram distribution of a contrast enhanced CTA image data and three major peaks, which correspond to the air in the lungs; blood filled regions and soft tissues can be observed from the histogram. To parameterize the histogram, a mixture of Gaussian model is used, and the model parameters, that is, the mean and variance for each Gaussian function, are determined by the application of Expectation Maximization (EM) method. Coronary arteries are vessels filled with blood; thus we assume that the intensities distribution derived within these vessels should be similar to the ones obtained from other blood-filled regions. Based on this assumption, we define the window size for each point on the active contour as follows:

(2) $$\begin{array}{c} r\left(x\right)=\min\_\text{scale}+\max\_\text{scale}\cdot \exp\left(\tau \cdot D\left(x\right)\right), \end{array}$$

where *r*(**x**) denotes the radius of the local window centered at **x** on the active contour and *τ* is a tuning parameter determining the decay speed of the exponential function. min_scale and max_scale are constants that can be defined based on prior knowledge about the maximum size of the vessel to be segmented; *D*_*B*_ represents the Bhattacharyya distance between the intensity distribution derived from the interior regions of the active contour within a local image and the blood filled regions. According to (2), it favors a large window size when the intensity distribution of current segmentation is similar to the one extracted from blood filled regions. On the other hand, if the intensity statistics of current segmentation deviate from the preassumed distribution, indicating that the localized image lacks contrast, a small window size would be selected. Since we utilize Gaussian model to approximate regional statistics in this paper, the Bhattacharyya distance measurement can be computed using the following simplified form:

(3) $$\begin{array}{c} D_{B}\left(p,q\right)=\frac{1}{4}\ln\left(\frac{1}{4}\left(\frac{\sigma_{1}^{2}}{\sigma_{b}^{2}}+\frac{\sigma_{b}^{2}}{\sigma_{1}^{2}}+2\right)\right)+\frac{1}{4}\left(\frac{\left(\mu_{1}-\mu_{b}\right)}{\sigma_{1}^{2}+\sigma_{b}^{2}}\right), \end{array}$$

where *μ*_1_,  *σ*_1_ denote the mean and variance calculated from the interior areas of the active contour within the local image, while *μ*_*b*_, *σ*_*b*_ are the mean and variance of the blood filled regions estimated from the Gaussian mixture model.

### 2.2. Nonparametric Shape Constraints

**Figure 3:**
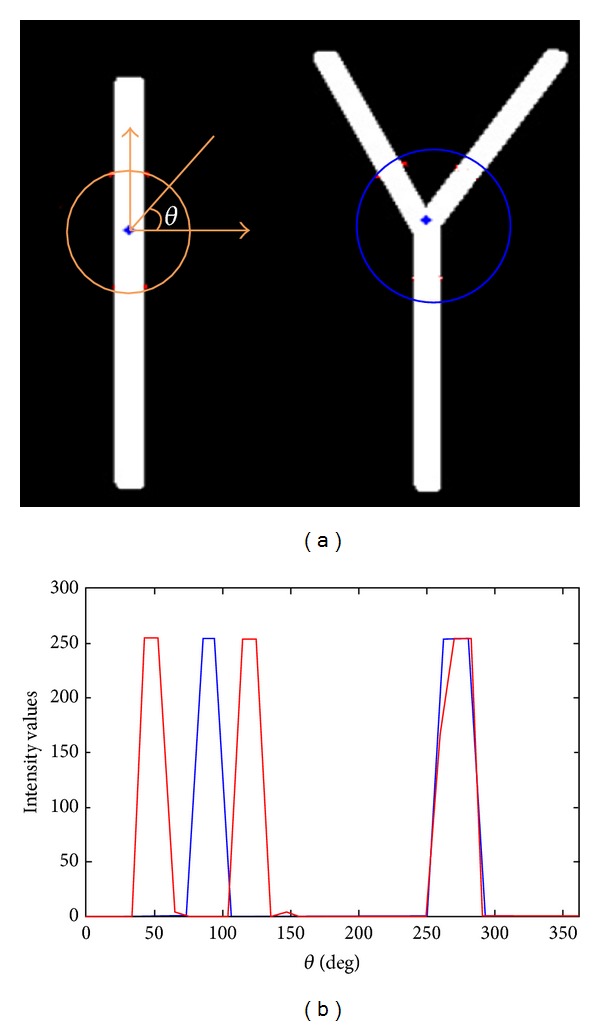
Synthetic image shows the intensity distribution along a circular sampled image profile for vascular structure. (a) The synthetic vessel image for straight and Y-shaped segments is depicted. (b) The intensity distribution along the circular sampled profile shown in (a) for straight vessel segment (blue) and vessel bifurcation (red), respectively.

**Figure 4:**
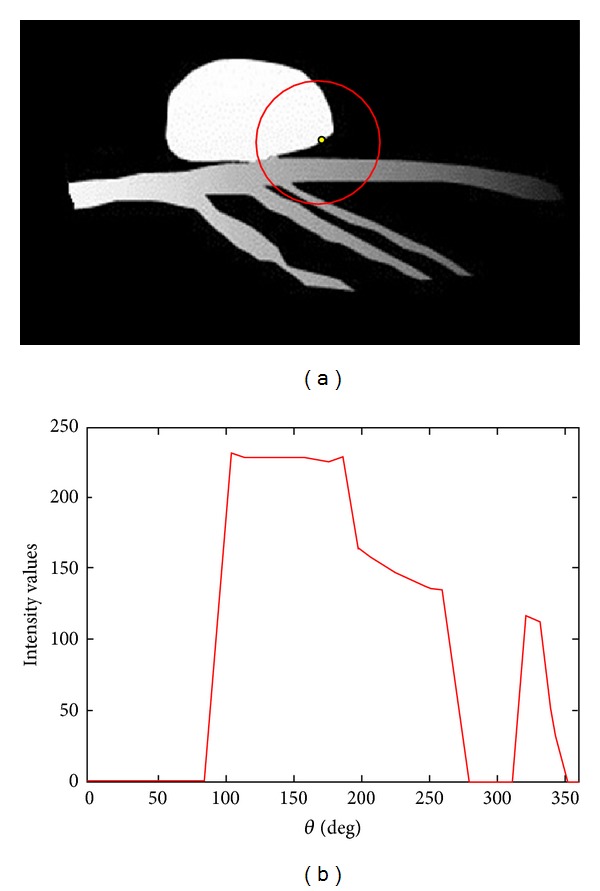
Synthetic vessel image illustrates the intensity distribution (shown in (b)) sampled along a circular profile (red circle in (a)) centered within the nonvascular structure.

Image segmentation based on intensity information alone is not able to always produce desired results since the boundaries between objects cannot be always characterized by image intensities. In this paper we propose incorporation of a nonparametric shape constraint into active contour framework, which prevents the contour deviated from being tubular structures. Motivated by the work reported by Qian and his coworkers [30], we use the intensity features calculated on a spherical/circular image profile to measure the likelihood of a pixel belonging to the vascular structures. As shown in Figure 3, the intensity distribution along the circular sampled profile has limited peaks for vascular structure. On the other hand, it can be observed that the probability density function sampled from the circular image profile for nonvascular structures is more dispersed (see Figure 4). Based on this observation, we define the nonparametric shape descriptor as

(4) v(x)=2H(x)max⁡⁡(H(x))−1, x∈inside(C(x)),

(5) H(x)=∫h(x,R,θ)·log⁡⁡h(x,r,θ)dθ,

where *H*(**x**) denotes the entropy of the profile, *h*(**x**, *r*, *θ*), sampled on a circular image profile, centered at **x** with radial *R*, and *θ* indicates the orientation of each sampling point on the image profile. It should be noticed that the radius of the circular sampling profile is not necessarily equal to the local window size *r*. *H*(**x**) is the entropy, a measurement of the randomness, of the intensity profile. The entropy will be large in magnitude if an intensity profile is evenly distributed, and, on the other hand, the value of *H*(**x**) approaches 0 when the sampled intensity is sparsely distributed (totally random). The maximum value of *H*(**x**) can be found when the sampled profile is equally distributed for all of the directions. *v*(**x**) defines the likelihood of a voxel being considered vascular structure based on the shape of current segmentation, which is normalised between −1 and 1. Since entropy is a computational consuming calculation, in this paper, we only perform the vesselness measurement within the interior region of the active contour. According to (4), if the shape of current segmentation deviates from being vascular, that is, *v*(**x**) → 1 for pixels inside the active contour, the shape based active contour energy would serve as an erosion term leading to inwards movement of the active contour. On the other hand, the shape based energy component would expand the active contour outwards for voxels with negative values of *v*(**x**).

### 2.3. Active Contour Energy

Let *C*(**x**) denote the active contour representing the boundaries of the object to be segmented. Given an image *I*(**y**) and the shape of current segmentation *v*(**y**), the posterior probability of a pixel being classified as belonging to the object can be calculated as

(6) P(y∈Ωi∩Ωx ∣ I(y),v(y)) =P(I(y),v(y) ∣ y∈Ωi∩Ωx)P(y∈Ωi∩Ωx)P(I(y)),

where *P*(**y** ∈ *Ω*_*i*_∩*Ω*_**x**_) denotes the prior probability of the a voxel being classified to region *Ω*_*i*_ among all the possible partitions in the local image *Ω*_**x**_. Assuming that the probabilities of a pixel being assigned to any partition are equal, we ignore this term to simplify our analysis. *P*(*I*(**y**)) represents the probability of the gray level values *I*(**y**), which can be neglected since it is independent of the segmentation of the image. *P*(*I*(**y**), *v*(**y**)) denotes the joint probability density distribution of the gray level value *I*(**y**) and the shape measurement *v*(**y**). If the gray level distribution of the image and the shape of the contour are assumed to be independent of each other, given the class of the pixel, the posterior probability about the intensity values and shape measurement for each voxel can be defined as

(7) P(I(y),v(y) ∣ y∈Ωi∩Ωx) =P(I(y)y∈Ωi∩Ωx)P(v(y)y∈Ωi∩Ωx).

The prior probability of *P*(*I*(**y**) | **y** ∈ *Ω*_*i*_∩*Ω*_**x**_) has been already defined in (1). Since the shape descriptor is only defined within the interior region of the active contour, that is, **y** ∈ *Ω*_1_, thus (7) can be written as

(8) P(I(y),v(y) ∣ y∈Ωi∩Ωx) ={P(I(y) ∣ y∈Ωi∩Ωx) × P(v(y) ∣ y∈Ωi∩Ωx),y∈Ω1P(I(y) ∣ y∈Ωi∩Ωx),y∈Ω2.

Maximizing the posterior probability in (6) is equivalent to minimizing its negative logarithm. Hence, for each given point **x** belonging to the contour *C*(**x**), the image-based energy *E*_**x**_ can be defined as

(9) Ex=−∑i=12∫Ωi∩Ωxlog⁡P(y∈Ωi∩Ωx ∣ I(y),v(y))dy=−∑i=12log⁡∫Ωi∩ΩxP(I(y),v(y) ∣ y∈Ωi∩Ωx)dy=−∑i=12[∫Ωi∩Ωxlog⁡P(I(y) ∣ y∈Ωi∩Ωx)dy] −λ∫Ω1∩Ωxlog⁡P(v(y) ∣ y∈Ω1∩Ωx)dy.

The prior probabilities, that is, *P*(*I*(**y**) | **y** ∈ *Ω*_*i*_∩*Ω*_**x**_) and *P*(*v*(**y**) | **y** ∈ *Ω*_1_∩*Ω*_**x**_), were defined in (1) and (4), respectively. *λ* is a weighting term compromising the contribution between the intensity based energy and the shape constraint.

Let *ϕ* be the signed distance function (i.e., the level set function) representing the active contour, and we assume that it takes positive values in the interior of the contour and is negative for regions outside of the contour; the active contour energy can be formulated as

(10) E=−∫H′(ϕ(x))  ×∑i=12{∫Ωi∩Ωx[log⁡P(I(y) ∣ y∈Ωi∩Ωx)]         ×Mi(ϕ(y))}dx +λ∫Ω1∩Ωx{log⁡P(v(y) ∣ y∈Ω1∩Ωx)         ×Mi(ϕ(y))dy}dx +μ∫|∇H(ϕ(x))|dx,

where *M*_1_(*ϕ*(**y**)) = *H*(*ϕ*) and *M*_2_(*ϕ*(**y**)) = 1 − *H*(*ϕ*), *H*(·) and *H*′(·) denotes the Heaviside and its first order derivative, respectively. The first two terms in the right hand side of (10) are the negative logarithm posterior probability defined in (7), and the minimums occur when the active contour is located on desired boundaries of the vessel while no leakages are detected. The last term is a smoothness regulator, which penalizes jagged edges by using the total length of the resulting boundaries. The constant *μ* controls the contribution of this smoothness term in the entire active contour functional. The associated Euler-Lagrange equation is defined as

(11) $$\begin{array}{c} \frac{\partial \phi }{\partial t}=\delta \left(\phi \right)\left(\mu \mathrm{div}\left(\frac{\nabla \phi }{\left|\nabla \phi \right|}\right)+\log\frac{p_{1}}{p_{2}}+\lambda \overset{}{\underset{\Omega_{1}\cap \Omega_{x}}{\int }}v\left(x\right)dy\right), \end{array}$$

where

(12) p1=∫Ω1∩Ωx12πσ1exp⁡⁡( −(μ1(x)−I(y))22σ12(x))dyp2=∫Ω2∩Ωx12πσ2exp⁡(−(μ2(x)−I(y))22σ22(x))dy

*p*_1_ and *p*_2_ represent the prior probability density distribution of the object and background, respectively. *δ*(·) denotes the Dirac delta function. The width of the localized kernel *r* is updated according to (2) at each iteration.

## 3. Experimental Results and Analysis

In this section, the proposed method is applied to both 2D synthetic and 3D real clinical images to demonstrate its efficiency. We firstly compare the proposed approach with a localized regional statistics based active contours model reported by Li et al. [24], to analyze the benefits introduced by the application of the adaptive scale scheme and shape constraint in terms of 2D synthetic images. The tuning parameters of the proposed technique were empirically determined and fixed throughout this experiment. In particular, we chose the smoothness weight *μ* at 0.2, and the shape based energy factor *v* was set to 0.4. The localized window size, *r*, is initialized as the maximum size of the vessel to be segmented. The width of the sampled circular profile is set to twice of the maximum size of the vessel of interest in order to ensure correct shape measurement for vessel bifurcations, and the number of sampled directions is 36 to balance its efficiency and robustness. The minimum and maximum scales for localized window are set to 4 and 10 voxels, respectively.

Three metrics, that is, true positive (TP), false positive (FP), and overlapping measurement (OM) [31], are employed to quantify the performance of the resulting segmentation produced by various methods, and their definitions are defined as follows:

(13) TP=NB∩NRNR,  FP=NB−NB∩NRNR,OM=2·NB∩NRNB+NR,

where the ground truth data *N*_*R*_ is defined as a binary image, with pixels labelled to one representing the object and zero for others and *N*_*B*_ represents pixels identified as the object by the vessel segmentation algorithms.

**Figure 5:**
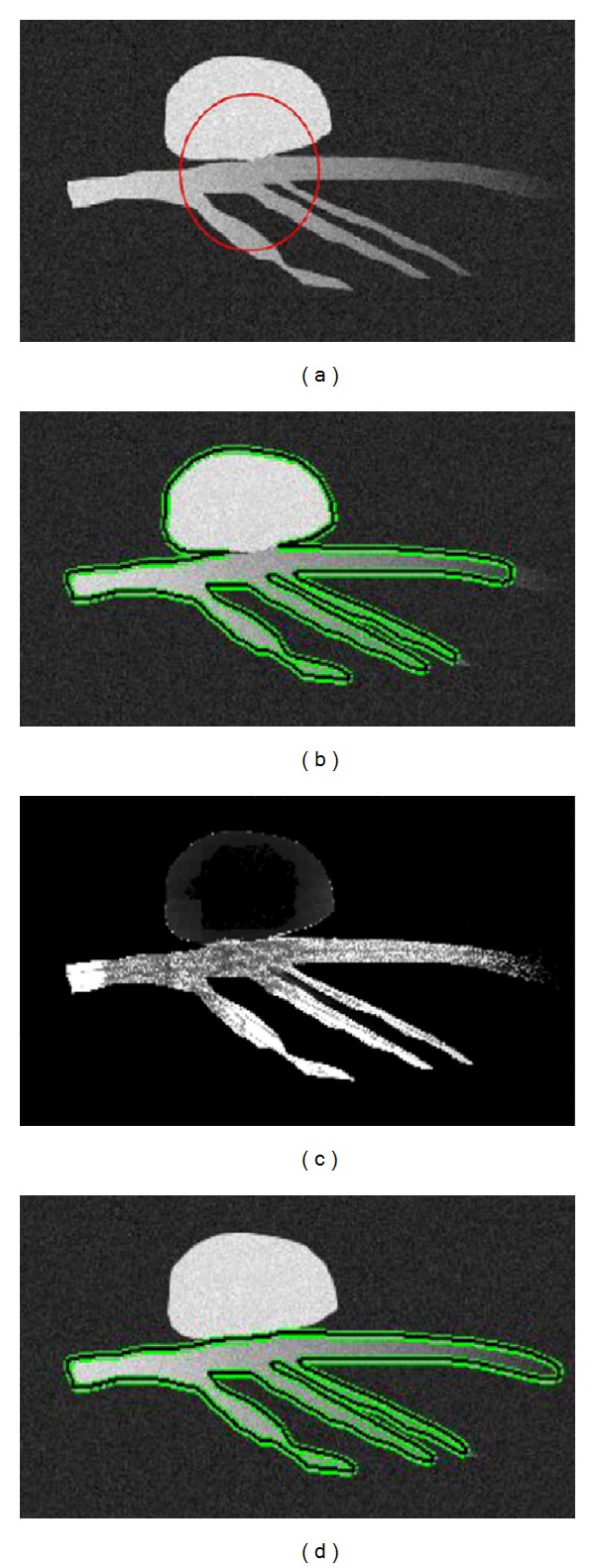
Segmentation results on 2D binary synthetic image. (a) Contour initialization shown as red circle, (b) results obtained using localized Chan-Vese method, (c) vesselness measurement calculated for each pixel within the vascular structure, and (d) illustration of the segmentation results from the proposed algorithm.

Figure 5(a) illustrates a 2D synthetic vessel image with additive Gaussian noise, and the active contour is initialized as a circle. In Figures 5(b) and 5(d), we show the segmentation results obtained by utilizing localized CV model in [24] and the proposed method, respectively.  Figure 5(c) is the proposed shape measurement calculated within the interior areas of the synthetic vessel, where bright pixels indicating such high possibilities considered as belonging to the vessel, while the dark ones imply that those pixels are more likely to be classified as nonvascular structures.

**Figure 6:**
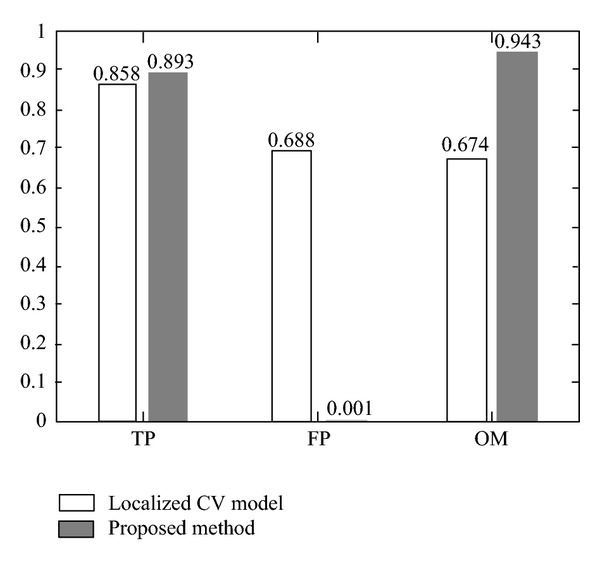
Comparison of the 2D synthetic image segmentation results obtained through the application of localized CV model and the proposed method.

The quantitative analysis of the resulting segmentations, in terms of the three metrics defined in (13), is presented in Figure 6. In general, it can be observed that the localized CV model is unable to extract the corrected vessel segment and leak into the proximal blob-like structure, which results in high FP rate and relatively smaller OM values. On the other hand, the proposed technique, combining intensity and shape information, greatly improves the segmentation results in terms of FP and OM metrics. In addition, by comparing Figures 5(b) and 5(d), we can find that the proposed method extracts more distal vessel segment than localized CV model, due to the application of the adaptive scale scheme.

Twelve coronary CT volumes were acquired from our calibrators, the mean size of the images is 512 × 512 × 282 with an average in-plane resolution of 0.40 mm × 0.40 mm, and the mean voxel size in the *z*-axis is 0.41 mm. The ground truth data were defined through manual delineation by trained biomedical student using interactive software developed in our lab. In this paper, three major branches of the coronary artery (i.e., the left and right coronary artery plus another largest branch among all of the coronary segments) are evaluated. Hausdorff distance, a measurement of the overlapping between surfaces, is utilized to evaluate the accuracy of these segmentation algorithms:

(14) $$\begin{array}{c} d_{H}\left(X,Y\right)=\max\left\{\underset{x\in X}{\sup}\;\underset{y\in Y}{\inf}d\left(x,y\right),\underset{y\in Y}{\sup}\;\underset{x\in X}{\inf}d\left(x,y\right)\right\}, \end{array}$$

where **X** and **Y** are the vertices of the mesh surfaces of the arteries corresponding to the segmentation results and the ground truth, respectively, and *d*(*x*, *y*) measures the Euclidean distance between points *x* and *y* belonging to vertices **X** and **Y**. The marching cube algorithm is used to construct the surface representation of the coronary arteries from the binary volume obtained from the segmentation/manual delineation.

**Figure 7:**
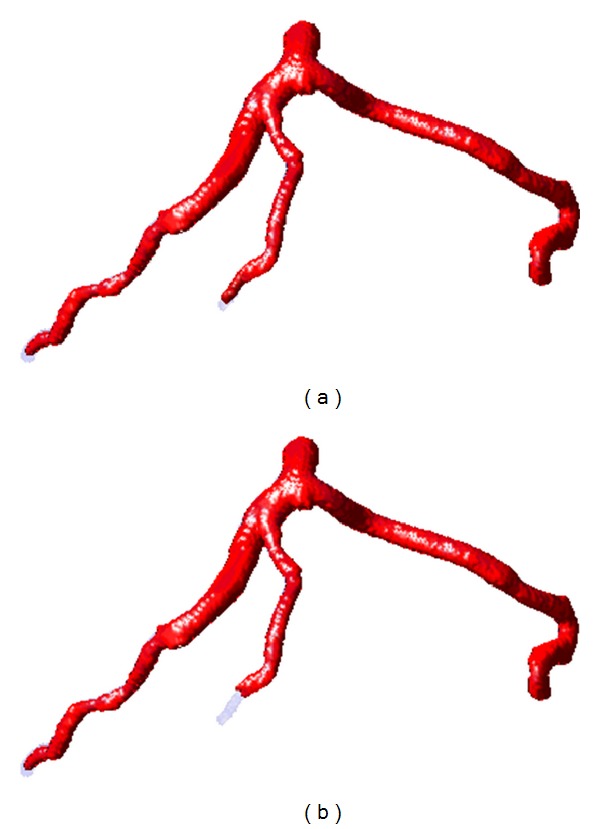
3D surface data illustrates the resulting segmentation (red surfaces) obtained from the proposed method (a) and localized CV model (b), respectively. Blue surface is obtained through manual delineation.

**Figure 8:**
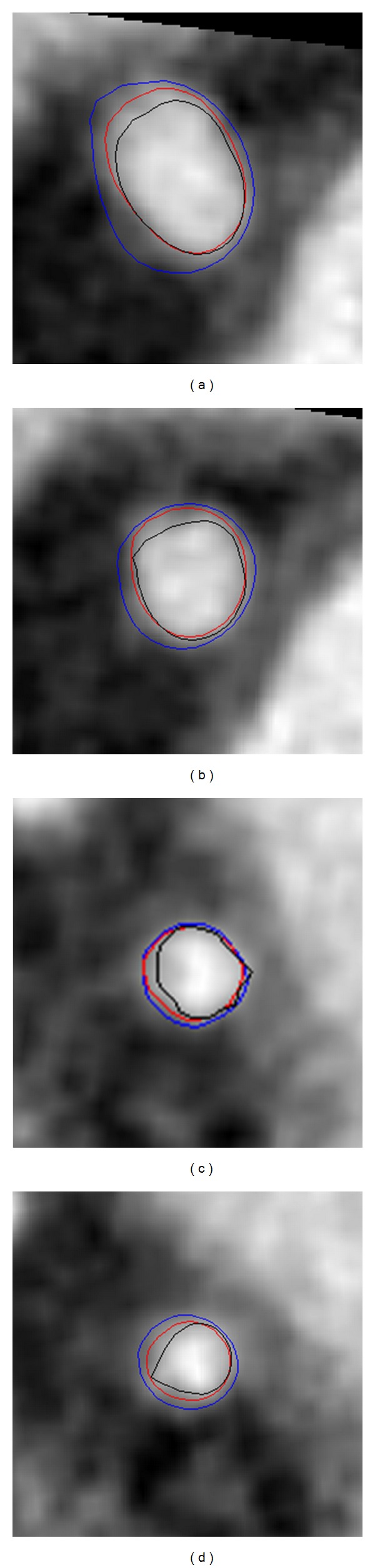
Comparison of the segmentation results in 2D axial images. (a) and (b) show the resulting segmentation on 2D cross-sectional images randomly taken from dataset number 5, while (c) and (d) are the resulting segmentation illustrated on 2D axial image picked from dataset number 9. The red contour represents the manually delineated reference standards, and the segmentation obtained from the proposed method and localized model are shown as blue and black contours, respectively. Note that these images are upsampled by a factor of 5 in order to increase the in-plane resolution.

**Figure 9:**
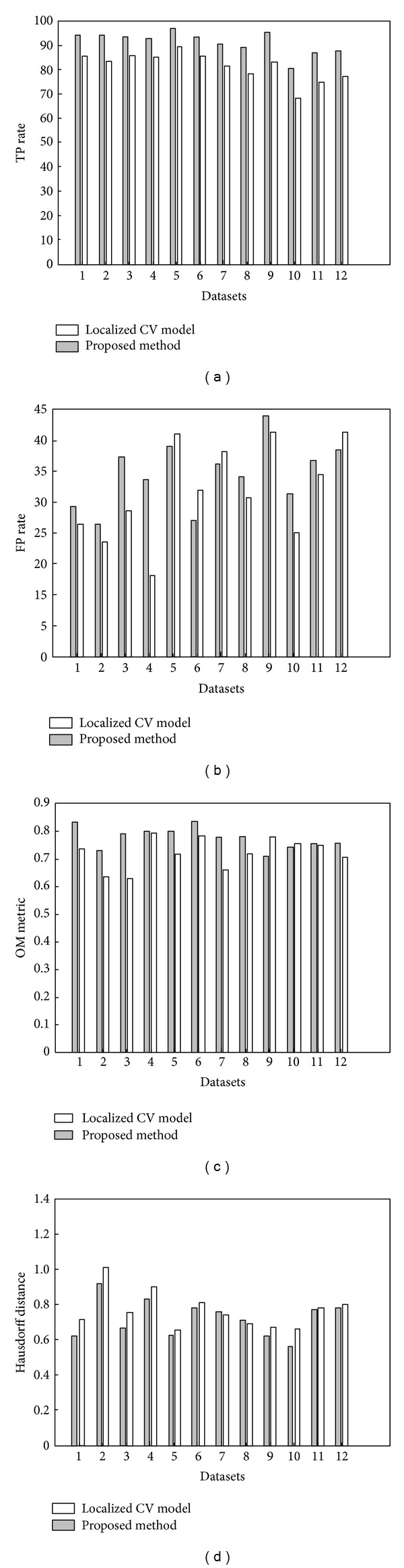
Bar plots depict the performance of the proposed technique and localized CV model, in terms of TP rate (a), FP metric (b), OM rate (c), and Hausdorff distance (d), on extraction of the coronary arteries in clinic CTA images.

In Figures 7 and 8, we present the segmentation results in terms of 3D surface image and 2D axial images of the coronary arteries, respectively. The quantification results are given in Figure 9. The tuning parameters of both of the two techniques were empirically determined and fixed throughout the experiments. Both the proposed algorithm and localized CV model were implemented in visual studio C++ 2008 on a standard desktop PC, and the average execution time was around 90 seconds for the proposed method to process a CTA dataset. On the other hand, localized CV model takes roughly 70 s to perform the same task.

As illustrated in Figure 9, the overall performance of the proposed method, in terms of TP rate and OM metric, is better than localized CV model, indicating the extraction of more details of the coronary arteries. Meanwhile, the value of FP measurement of the proposed approach is higher than the localized CV algorithm, which implies that the proposed method tends to oversegment the vessels. This observation is also demonstrated in Figure 8, where we illustrate the resulting segmentation on 2D axial image as contours. In these images, the red contours denote manually delineated ground truth, and the segmentations obtained from the proposed model and localized CV method are shown as blue and black curves, respectively. As the proposed method employs an adaptive scale selection scheme, it increases the capability of the proposed method in defining relatively weak edge, indicating higher OM and TP rates. In addition, the proposed method outperforms localized CV model in terms of Hausdorff distance metric, implying that the proposed method is able to more precisely extract the coronary arteries with subvoxel accuracy.

## 4. Conclusion and Future Work

Precise and robust segmentation of vascular structures plays a vital role in clinical tasks, such as stenosis grading and functional analysis of blood circulations. In this paper, we presented novel algorithm for the segmentation of coronary arteries in 3D CTA images based on the framework of active contours. Compared with conventional local regional statistics based models, the proposed method enables the extraction of more detailed vessels by the introduction of an adaptive scale selection scheme. The leakage problem, which cannot be identified by using intensity information alone, was solved by using a nonparametric shape constraint. Experimental results on both synthetic and real clinical datasets demonstrated the efficiency and robustness of the proposed model in defining the correct boundaries of vascular structures. In terms of future research, we aim to identify and segment the soft plaques within the coronary arteries, which is a more challenging task due to the lack of appropriate intensities features.
